# Hydroxytyrosol protects isoproterenol-induced myocardial infarction through activating notch signaling

**DOI:** 10.22038/ijbms.2024.81495.17637

**Published:** 2025

**Authors:** Elif Onat, Ahmet Türk, Nevin Kocaman, Serhat Hançer, Solmaz Susam, Ali Parlar, Selin Turhan, Mehmet Kaya Özer

**Affiliations:** 1 Department of Medical Pharmacology, Faculty of Medicine, Adıyaman University, Adıyaman, 02040, Turkey; 2 Department of Histology and Embryology, Faculty of Medicine, Adıyaman University, Adıyaman, 02040, Turkey; 3 Department of Histology and Embryology, Faculty of Medicine, Fırat University, Elazığ, 23119, Turkey; 4 Department of Medical Biochemistry, Faculty of Medicine, Adıyaman University, Adıyaman, 02040, Turkey

**Keywords:** DLL4, Hes1, Hydroxytyrosol, Myocardial infarction, Notch1

## Abstract

**Objective(s):**

In this investigation, the protective effects of hydroxytyrosol (HT) administered prior to myocardial infarction in rats were examined, with a particular focus on its potential roles within the Notch pathway.

**Materials and Methods:**

The animals were categorized into seven groups (n=7): control, myocardial infarction (MI) 6^th^ hr, MI 24^th^ hr, MI 7^th^ day, MI+HT 6^th^ hr, MI+HT 24^th^ hr, MI+HT 7^th^ day. In order to create infarction, the rats received a subcutaneous injection of isoproterenol at a dose of 200 mg/kg. Rats were given 4 ml/kg/day liquid containing HT orally for six weeks before infarction. Histopathological examination was conducted on heart tissue to assess Notch1, Hes1, and DLL4. Biochemical parameters were analyzed in serum using the ELISA method.

**Results:**

The study revealed an increase in Notch1 and DLL4 levels, particularly at the 24^th^ hr and 7^th^ day after the occurrence of myocardial infarction. DLL4 increased at 24 hr and 7 days of infarction after HT administration compared to control. Hes1 levels increased towards the seventh day after infarction and following HT application before infarction. It was noted that the severity of histopathological damage in heart tissue was reduced at the 24^th^ hr of infarction in rats treated with HT prior to infarction. A significant decrease in fibrosis was observed on the seventh day of infarction in rats given HT before infarction. The levels of biochemical parameters decreased with the administration of HT before the occurrence of infarction.

**Conclusion:**

HT is thought to exert a cardioprotective effect in MI, potentially mediated through the Notch pathway.

## Introduction

In the last ten years, there has been a notable increase in the prevalence of cardiovascular diseases, becoming the primary cause of global mortality (1). Acute myocardial infarction (AMI) is characterized by the necrosis of cardiomyocytes due to prolonged myocardial ischemia, disrupting the balance between myocardial demand and coronary blood flow (2). AMI is linked to an inflammatory response initiated by changes in the extracellular matrix, attracting proteolytic enzymes and free radicals towards the remodeled myocardium (2). This inflammatory process has the capacity to impact the magnitude of myocardial lesions, and the application of anti-inflammatory medications could potentially alleviate the occurrence of ischemic lesions (3). Moreover, the utilization of anti-oxidants in therapy may demonstrate protective effects on the heart by diminishing oxidative stress in instances of myocardial ischemia and reperfusion injury (4). 

The significant protective effects of olive oil are attributed to its phenolic compounds and fatty acid composition. Among these phenolic compounds is hydroxytyrosol (HT), a molecule known for its anti-oxidant activity (IUPAC name: 4-(2-Hydroxyethyl)-1,2-benzenediol). HT can be obtained from various sources, including olive leaves and olive oil. It maintains stability in its free form and demonstrates facile penetration into tissues (5). It is assumed that HT exerts cardioprotective, neuroprotective, anticarcinogenic, antimicrobial, and significant endocrine and biological effects (6). Despite extensive investigation, the precise molecular mechanisms responsible for numerous of these effects remain incompletely understood.

The Notch signaling pathway is recognized for its crucial involvement in the development of the mammalian heart. Notch1 is thought to be present at low levels in the postnatal myocardium and at high levels in the immature myocardium in embryonic heart development. Notch1, Hes1, and DLL4 levels are very low at birth in the heart. However, it has been observed to increase significantly in cardiomyocytes four days after myocardial infarction (MI) (7), indicating that the Notch pathway may have roles in regulating myocardial damage. Earlier investigations have indicated that the activation of Notch signaling prompts the differentiation of stem cells and the formation of new blood vessels, participating in the recovery from myocardial ischemic injury by reducing myocardial fibrosis and improving cardiac function (8-13). In various investigations, the activation of the Notch pathway has been shown to mitigate the impact of myocardial ischemia and enhance myocardial function following myocardial infarction (14). In addition, it is also known that the Notch pathway promotes cardiac regeneration by improving angiogenesis and is associated with recovery of MI by reducing apoptosis, fibrosis, and oxidative stress (15-19). 

Although it is known that olive oil and phenolic compounds have cardioprotective effects, the mechanisms of this protective effect have not been fully clarified. Therefore, while delving into the cardioprotective potential of HT, a constituent of olive oil, the study sought to examine the possible involvement of the Notch pathway, which is considered to play a significant role in the pathophysiology of MI.

## Materials and Methods

***Animals and experimental design***

The Animal Ethics Committee of Adıyaman University approved the study protocol (Protocol no: 2023/026). The experiments were performed according to the “Guide for the Care and Use of Laboratory Animals”. A total of 49 Sprague-Dawley male rats (weighing 200-250g, 8-10 weeks old) obtained from Adıyaman University Experimental Research Center were utilized, and the standard water and feed *ad libitum* was given to animals. The rats were distributed into seven groups (n: 7): Group-I (Control), Group-II (MI 6th hour), Group-III (MI 24^th^ hr), Group-IV (MI 7^th^ day), Group-V (MI+HT 6^th^ hr), Group-VI (MI+HT 24^th^ hr), and Group-VII (MI+HT 7^th^ day) (20, 21). No procedures were made for the control group. Kale Naturel Herbal Products Company in Turkey provided HT in liquid form. From this solution containing HT, rats in Groups V, VI, and VII received an oral administration of 4 ml/kg/day for 6 weeks (22). The rats were induced with myocardial infarction by subcutaneously administering 200 mg/kg of ISO (Isoproterenol hydrochloride, I5627, Sigma-Aldrich Corporation, St. Louis, USA) (20, 21). The rats underwent intraperitoneal anesthesia through a mixture of ketamine (75 mg/kg) and xylazine (10 mg/kg). Subsequently, blood samples were extracted from the hearts of the rats in Group-II and V at 6 hr, in Group-III and VI at 24 hr, and in Group IV and VII on the seventh day. Serum samples were stored at −80 °C to perform biochemical studies. Following the conclusion of the experiment, for histological examinations, the tissues from the heart were preserved in a 10% formaldehyde solution.

***Serological analyses***

Serum CK-MB and Troponin I levels were assessed using an immunoassay analyzer (AQT90 FLEX; Radiometer, Copenhagen, Denmark). For AST and LDH levels, analysis was conducted utilizing the Architect c8000 Chemistry System (Abbott Diagnostics) and commercially available kits from Abbott Diagnostics.

***Histochemical examination***

The heart tissues of the rats underwent standard histological processing and were encased in paraffin blocks. Sections measuring 5 µm in thickness were derived from these blocks, and various staining techniques, including Masson Trichrome, Hematoxylin and Eosin, and Immunohistochemical stains, were employed.

***Immunohistochemical examination***

Immunohistochemical procedures were carried out following the protocols outlined by Kocaman and Artas (23). Immunohistochemistry investigations were made with 3μm-thick histological tissue microarray slides by using: Notch1, Hes1, and DLL4 polyclonal antibodies (respectively= 1:200, Cat #= Fnab05799, Fine test; Cat #= BTAP03944, BT-Lab, Fine Test; 1:500, Cat #= Fnab02416). The samples were assessed and photographed using the Zeiss Axio Scope A1 microscope (Carl Zeiss Microscopy GmbH, 07745 Jena, Germany). Subsequently, a histoscore for Notch1, Hes1, and DLL4 was established based on the results of immunohistochemical staining.

Evaluation of staining intensity under the microscope involved assigning values based on the following criteria: “0” for negatively stained areas, “0.1” for areas with less than 25% staining, “0.4” for areas with 26–50% staining, “0.6” for areas with 51–75% staining, and “0.9” for areas with near homogeneous staining (76–100%). The computation of the ultimate histoscore involved applying the specified formula, which combines the parameters of distribution and intensity.

Histoscore = Distribution × Intensity (23)

***Statistical analysis***

SPSS statistical program (version 22, IBM Corporation, USA) was utilized for the statistical calculations. The One-Way ANOVA Test was employed, and subsequent post-hoc multiple comparisons were carried out by using the Tukey HSD test. To assess normal distribution, the Kolmogorov-Smirnov test was performed. The results are expressed as mean ± SD, with statistical significance considered at *P*<0.05.

**Table 1:** Comparison of ELISA results of serum between the each group of rats

Groups	AST (U/l)	LDH (µ/l)	CK-MB (U/l)	Troponin I (µg/l)
Control	149± 43.35	438.75± 217.97	687.46± 128.48	0.31± 0.42
MI 6^th^ hr	442.12± 42.95 a	1301.2± 273.88 a	1226.1± 189.12 a	11.07± 2.46 a
MI 24^ th^ hr	389.25± 50.28 a	1246.1± 323.18 a	1118.7± 165.97 a	9.21± 3.99 a
MI 7th day	250.38± 21.19 a	1053.9± 128.64 a	951.51± 119.78	7.2± 0.72 a
MI+HT 6^ th^ hr	328.75± 37.83 ab	871.25± 140.45 ab	873.43± 153.35 b	7.21± 2.03 ab
MI+HT 24^ th^ hr	274± 27.19 ac	796.12± 79.92 c	757.37± 135.98 c	5.18± 0.87 ac
MI+HT 7^ th^ day	163.88± 28.48 d	645.12± 74.43 d	678.25± 101.21 d	3.06± 0.87 d

Error bars indicate SD. a. *P*<0.05 compared with control. b. *P*<0.05 compared with MI 6^th^ hr. c. *P*<0.05 compared with MI 24^th^ hr. d. *P*<0.05 compared with MI 7^th^ day

MI: Myocardial infarction; MI+HT: Myocardial infarction+Hydroxytyrosol; AST: Aspartate aminotransferase; LDH: Lactate dehydrogenase; CK-MB: Creatine kinase-MB

**Figure 1:**
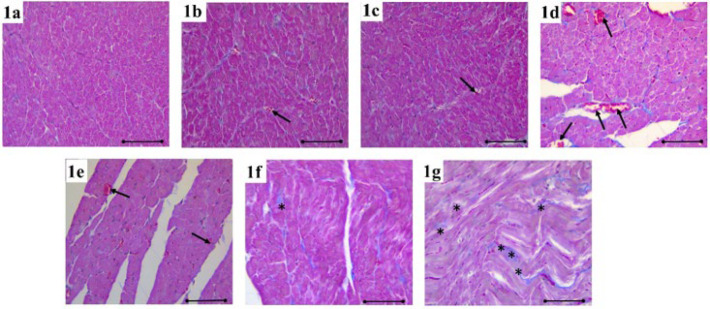
In the heart tissue stained with Masson’s trichrome, congestion (black arrow), mononuclear cell increase (red star), fibrosis (black star), and degeneration in cardiac muscle cells (red arrow) were shown in the each group of rats

Scale bar: 100 µm. 1a. control, 1b. MI 6^th^ hr, 1c. MI+HT 6^th^ hr, 1d. MI 24^th^ hr, 1e. MI+ HT 24^th^ hr, 1f. MI 7^th^ day, 1g. MI+HT 7^th^ day.

**Figure 2:**
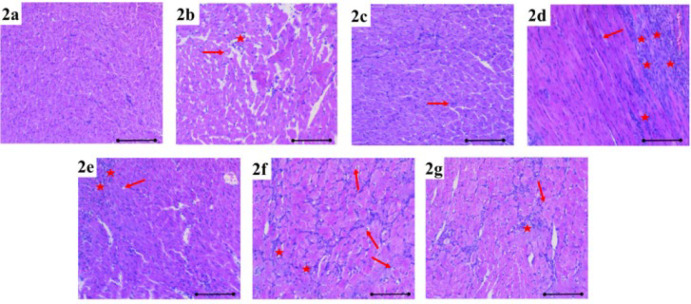
Mononuclear cell increase (red star) and degeneration in cardiac muscle cells (red arrow) were observed in heart tissue stained with Hematoxylin and Eosin in the each group of rats

Scale bar: 100 µm. 2a. control, 2b. MI 6^th^ hr, 2c. MI+HT 6^th ^hr, 2d. MI24^th^ hr, 2e. MI +HT 24^th^ hr, 2f. MI 7^th^ day, 2g. MI+HT 7^th ^day

**Figure 3:**
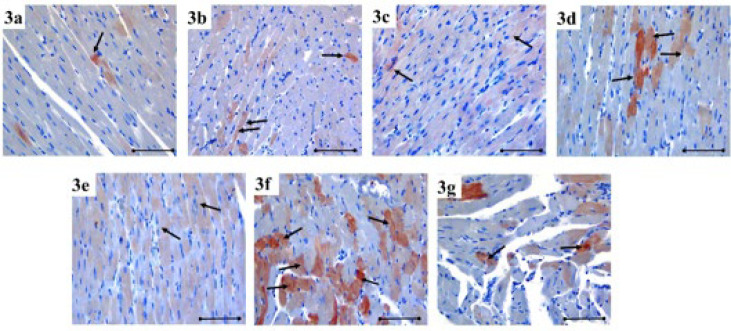
Notch1 immunoreactivity in heart tissue is demonstrated by immunohistochemical staining (black arrow) in the each group of rats

3a. control, 3b. MI 6^th^ hr, 3c. MI+ HT 6^th^ hr, 3d. MI 24^th^ hr, 3e. MI+HT 24^th^ hr, 3f. MI 7th day, 3g. MI+HT 7^th ^day. AEC chromogen, Mayer hematoxylin. Scale bar: 100 µm.

**Figure 4:**
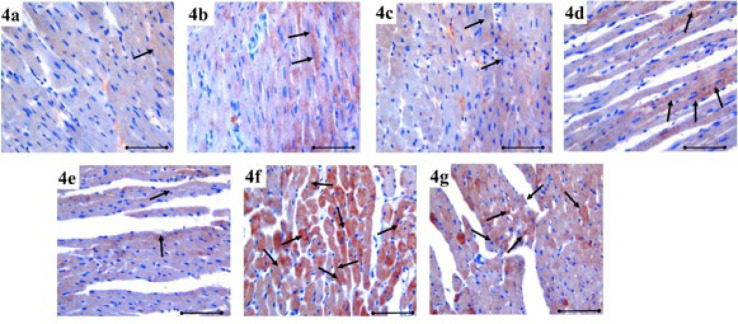
Hes1 immunoreactivity in heart tissue is demonstrated by immunohistochemical staining (black arrow) in the each group of rats

4a. control, 4b. MI 6th hr, 4c. MI+HT 6^th^ hr, 4d. MI 24^th^ hr, 4e. MI+ HT 24^th ^hr, 4f. MI 7^th^ day, 4g. MI+HT 7^th^ day. AEC chromogen, Mayer hematoxylin. Scale bar: 100 µm.

**Figure 5:**
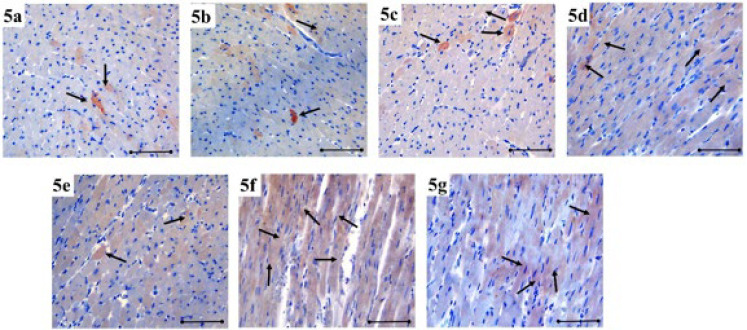
DLL4 immunoreactivity in heart tissue is demonstrated by immunohistochemical staining (black arrow) in the each group of rats

5a. control, 5b. MI 6^th^ hr, 5c. MI+ HT 6^th^ hr, 5d. MI 24^th^ hr, 5e. MI+ HT 24^th^ hr, 5f. MI 7^th^ day, 5g. MI+HT 7^th^ day. AEC chromogen, Mayer hematoxylin. Scale bar: 100 µm.

**Figure 6:**
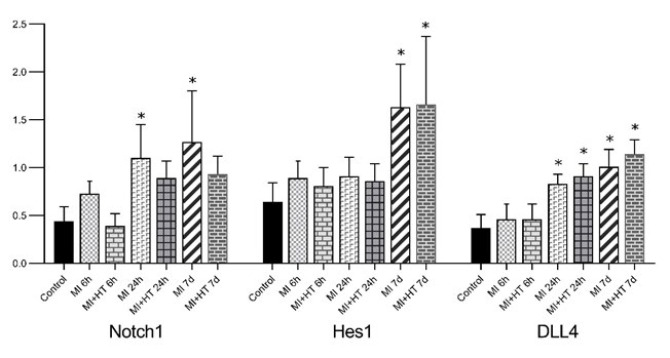
Immunohistochemical findings for Notch1, Hes1, and DLL4 in heart tissues in the each group of rats

Error bars indicate SD. *P<0.05 compared with control. MI: Myocardial infarction; MI+HT: Myocardial infarction+Hydroxytyrosol

## Results

***Biochemical findings***

After myocardial infarction, significant increases were observed in AST, LDH, CK-MB, and Troponin I levels compared to the control group at 6, 24 hr, and seven days (*P*˂0.001). AST levels were found to be significantly lower in the MI groups receiving HT than in the MI groups at 6, 24 hr, and seven days after MI (*P*˂0.001, *P*˂0.001, *P*=0.005, respectively). Likewise, LDH levels were observed to be significantly lower in the MI groups receiving HT at 6, 24 hr, and seven days after MI (*P*=0.011, *P*=0.007, *P*=0.019, respectively). CK-MB levels were found to be lower in the MI groups receiving HT at the 6th, 24^th^ hr, and seventh days of MI (*P*=0.003, *P*=0.002, *P*=0.043, respectively). Troponin levels were observed to be lower in the MI groups receiving HT compared to the MI groups at the 6^th^, 24^th^ hr, and seventh days of MI (*P*=0.037, *P*=0.026, *P*=0.019, respectively) (Table 1).

***Histochemical findings***

Following the investigation of Masson trichrome and Hematoxylin and Eosin (H&E) staining of all groups under light microscopy, the heart tissue of the rats in the control group appeared normal (Figure 1a, Figure 2a**)**. Compared to the control group, mild congestion (black arrow) was observed in the MI 6th hour (Figure 1c, Figure 2b) and MI+HT 6th hour groups (Figure 1b, Figure 2c), while mononuclear cell increase (red star) and degeneration of cardiac muscle cells were observed in the MI 6th hour (red arrow). When compared to the control group, congestion and mononuclear cell increase were observed in the MI 24^th^ hour group (Figure 1e). However, although the damage to the heart tissue was greater in the MI+HT 24^th^ hr group (Figure 1e, Figure 2d) compared to the control group, the histopathological damage severity was found to be lighter compared to the MI 24^th^ hour group. 

Considerable fibrosis (indicated by the black star) was evident in the MI 7^th^ day group (Figure 1f, Figure 2f) in contrast to the control group. A noteworthy reduction in fibrosis was noted in the MI+HT 7th day group (Figure 1g, Figure 2g) compared to the MI 7th day group.

***Immunohistochemical findings***

Upon examination of immunohistochemical staining for Hes1, Notch1, and DLL4 immunoreactivity in cardiac tissue under light microscopy, the subsequent findings were obtained.

Notch1 immunoreactivity increased in MI, especially at 24 hr and on the seventh day (*P*=0.008). Compared to the control group, Notch1 immunoreactivity increased at 24 hr and on the seventh day of MI after HT administration, but this was not at a significant level (Figure 6**)**. Immunoreactivity histoscores of Notch1 for all groups are given in Figure 3.

Hes1 immunoreactivity increased more significantly on the seventh day of MI (*P*=0.001). Hes1 immunoreactivity increased compared to the control group more significantly on the 7^th^ day of MI after HT administration (*P*=0.001) (Figure 6). The immunoreactivity histoscores of Hes1 for all groups are given in Figure 4.

DLL4 immunoreactivity increased in MI, especially at 24 hr and on the seventh day (*P*˂0.001). DLL4 immunoreactivity increased at the 24^th^ hr and on the seventh day of MI after HT administration compared to the control group (*P*˂0.001) (Figure 6). The immunoreactivity histoscores of DLL4 for all seven groups are given in Figure 5.

## Discussion

Polyphenols are increasingly gaining acceptance as therapeutic agents in a variety of conditions, such as atherosclerotic and cardiovascular diseases. Multiple investigations have highlighted a negative relation between the risk of cardiovascular disease, mortality, and the intake of polyphenols. Within these biologically active compounds, HT derived from olives stands out for its anti-oxidative, antiatherosclerotic, cardioprotective, neuroprotective, anticancer, and various other effects (24). In this study, backed by biochemical and histological evidence, it was determined that HT exhibits a cardioprotective effect when administered before MI. Consequently, this study provided novel insights by revealing that the Notch signaling pathway, renowned for its protective function in the cardiovascular system, might play a role in this particular effect for the first time.

Being the primary polyphenolic constituent in olive oil and a robust neutralizer of various free oxygen radicals, HT demonstrates defensive properties shielding cells from the deleterious effects of oxidative stress (25). It was demonstrated in both *in vitro* and *in vivo* investigations that HT exhibits higher activity compared to synthetic anti-oxidants. Furthermore, it can stimulate anti-oxidant enzymes, thereby enhancing the endogenous defense system (24-28). HT (20 mg/kg) has been found to protect heart function by decreasing MI size, oxidative stress, and cardiomyocyte apoptosis, through the phosphatidylinositol-4,5-biphosphate 3 kinase (PI3K)/protein kinase B (Akt) signal pathway in adult Sprague Dawley male rats in myocardial I/R injury (29). In another study, in the myocardial I/R model in rats, when isolated hearts were treated with various doses (10, 100, and 1,000 µM) of HT 10 min before ischemia, there was not any effect for 10 µM HT. It was revealed that treatment with HT at 100 or 1.000 µM reduced myocardial infarction area and myocardial damage in rats by inhibiting MPTP opening. Moreover, there was a notable distinction observed in the cardiac safeguarding conferred by 100 and 1,000 µM HT (30). Consequently, it has been proposed that the dose-dependent response to HT manifests at 10–100 µM, consistent with observations from prior research by Pan *et al.* (31). In our study, the elevation of blood plasma levels of LDH, Troponin-I, CK-MB and AST, as well as congestion, degeneration, fibrosis, and mononuclear cell, increased in myocardial cells with ISO application was observed. After HT application before MI, pathological changes in the heart tissue were alleviated, especially in fibrosis. The elevation of blood serum levels of LDH, CK-MB, AST, and troponin I, typically associated with myocardial infarction, showed a decrease following HT administration. These findings once again demonstrate the cardioprotective effect of HT (22).

Several publications have highlighted the protective role of the Notch1 pathway in averting myocardial fibrosis. Notably, mice lacking Notch1 displayed myocardial fibrosis following myocardial injury in contrast to their wild-type counterparts. Additionally, heightened Notch1 activity mitigates TGF-β1/SMAD3 signaling, impeding the conversion of fibroblasts into myoblast fibroblasts (12). Various research, encompassing both laboratory and animal studies, provides compelling evidence for the significant involvement of the Notch pathway in diminishing cardiomyocyte apoptosis (18). Specifically, Notch1 was identified as a key regulator of apoptosis, modulating the expression of Bax, Bcl-2, caspase-3, and caspase-9 in a hypoxic cardiomyocyte model (32). Moreover, in murine models of MI, the Notch pathway demonstrated a protective effect against apoptosis by coordinating the activity of the transcription factor RBP-J (32). In a separate study, it was observed that Notch1 inhibits apoptosis by impeding the binding of NF-κB to DNA, serving as a negative regulator to enhance cell survival (33). Nevertheless, certain studies have suggested that the Notch pathway has a role in anti-oxidative stress (10, 34, 35). For instance, investigations have highlighted the potential of a TNF-α inhibitor in suppressing oxidative stress during myocardial I/R injury, and this effect is, at least in part, attributed to the modulation of Notch1 signaling (35). Given the complex interconnection between the Notch pathway and anti-oxidative stress, researchers have devised therapeutic strategies and utilized stem cells to enhance Notch1 signaling to mitigate oxidative stress (11, 34). For example, the overexpression of Aldolase A (ALDOA), resulting in the up-regulation of VEGF/Notch1/Jagged1, has demonstrated effectiveness in reducing oxidative stress and apoptosis induced by hypoxia/reperfusion in cardiomyocytes (34).

In another study, extracellular vesicle-cardiac mesenchymal stem cells (EV-C-MSCs) carrying an exogenous intracellular domain (NICD) promoter were utilized to reduce apoptosis of cardiomyocytes and endothelial cells in *in vitro* oxidative stress and ischemia (11).

In addition, it has been confirmed that the Notch pathway responds to hypoxia, and there are intricate interactions, both direct and indirect, between Notch pathway molecules and the hypoxia-inducible factor (HIF) pathway (36). In the initial stages, the activation of the Notch pathway is triggered by hypoxia, where the gradual buildup of HIF stimulates the Notch signaling pathway in tissues. This activation results in the expression and synthesis of the NICD promoter, initiating the expression of downstream genes like Hes1 and Hey2 (37). Moreover, there is a collaborative interaction between the Notch pathway and hypoxia. For instance, myocardial ischemia triggers the activation of the Notch signaling pathway, induces HIF expression, alleviates myocardial I/R injury, and leads to the expression of the target gene Hes1 (38).

Additionally, the HIF-1α-Notch1 pathway is crucial for generating arterial endothelial cells, facilitating arteriogenesis, and revascularizing ischemic tissue (**37**). This collaborative effect between HIF-1α and Notch signaling pathways optimally enhances the recovery of the damaged myocardium. In our study, in alignment with these findings, the observed tendency of Notch1, Hes1, and DLL4 levels to increase in heart tissue, especially towards the seventh day of MI induced by isoproterenol (ISO) in rats, once again indicates the activation of this pathway.

The C-Met/HGF and PI3K/Akt pathways have been reported to trigger the activation of Notch signaling following myocardial injury. Notably, in the adult myocardium post-myocardial injury, not only does Notch signaling initiate activation, but it also augments the expression of the PI3K/Akt pathway. This reciprocal relationship suggests a positive feedback mechanism that promotes survival between Notch and Akt signaling (14). The protective impact of HT on heart function, encompassing the reduction of myocardial infarct size, oxidative stress, and cardiomyocyte apoptosis, underscores its significance through (PI3K)/protein kinase B (Akt) pathway in myocardial I/R damage and that the Notch pathway has a positive interaction with (PI3K)/protein kinase B (Akt) signaling pathway in the protective effect of HT in MI suggests that two signaling pathways play roles in the protective effect of HT. In our study, the tendency of Notch signaling members to increase towards the seventh day in rats with MI after HT administration shows once again that this pathway may be effective in the protective effect of HT.

This present study has inherent limitations that warrant cautious interpretation of the findings and the need for future research. Integration of imaging techniques like ECG/ECO could enhance the study’s comprehensiveness. Further exploration is essential to delineate the clinical relevance of HT in individuals with coronary heart disease. Identification of additional signaling components contributing to HT-induced myocardial protection is imperative. Robust, long-term investigations with larger sample sizes are imperative to evaluate the potential positive effects of HT on the survival of rats undergoing myocardial I/R.

## Conclusion

It is hypothesized that the Notch pathway may play a role in HT’s cardioprotective effect. Biochemical and histopathological evidence supports this hypothesis, demonstrating HT’s potential protective effect in MI.

## Declaration

We have not used any AI tools or technologies to prepare this manuscript.
